# Insufficient Evidence to Ascertain the Long-Term Survival of PEEK Dental Prostheses: A Systematic Review of Clinical Studies

**DOI:** 10.3390/polym14122441

**Published:** 2022-06-16

**Authors:** Zohaib Khurshid, Binoy Mathews Nedumgottil, Ramy Moustafa Moustafa Ali, Sompop Bencharit, Shariq Najeeb

**Affiliations:** 1 Department of Prosthodontics and Dental Implantology, College of Dentistry, King Faisal University, Al-Ahsa 31982, Saudi Arabia; bmuottil@kfu.edu.sa (B.M.N.); rmmali@kfu.edu.sa (R.M.M.A.); 2 Department of Removable Prosthodontics, Faculty of Dentistry, Fayoum University, Fayoum 2933110, Egypt; 3 VCU Philips Institute for Oral Health Research, Department of Biomedical Engineering, School of Dentistry, College of Engineering, Virginia Commonwealth University, Richmond, VA 23298, USA; sbencharit@vcu.edu; 4 Schulich School of Medicine and Dentistry, University of Western Ontario, London, ON N6A 3K7, Canada; snajeeb3@uwo.ca

**Keywords:** polyetheretherketone, dental prostheses, prosthodontics, dental implants, obturators

## Abstract

Introduction: Polyetheretherketone (PEEK) is a polymer that is used in the construction of orthopaedic and dental implants. It is also used to construct removable and fixed dental prostheses due to its superior mechanical and esthetic properties compared to conventional materials. This systematic review aims to analyse and appraise the literature concerning PEEK dental prostheses critically. Methods: The following focused question was constructed ‘Are dental prostheses made of PEEK inferior to those made of other materials in terms of clinical- and patient-reported outcomes?’. The CONSORT (Consolidated Standards of Reporting Trials) tool was used for the quality assessment of the randomised clinical trials. The STROBE (Strengthening the Reporting of Observational Studies in Epidemiology) quality assessment tool was used to assess the quality of observational studies and the case reports were evaluated using the CARE (Case Report) guidelines. Results: A total of 12 studies were included in this review. Two case studies received an overall grade of medium and the overall quality of six studies was graded as ‘low’. All three observational studies and the only randomised controlled trial received scores of ‘medium’. Conclusion: PEEK-based dental prostheses may provide a viable and more esthetic alternative to conventional prosthodontic appliances. However, within the limitations of this study is the evidence to ascertain the long-term viability of PEEK-based dental prostheses. Future studies should focus on conducting large-scale, multicenter trials to compare the survival rate of PEEK prostheses to that of conventionally available prosthodontic appliances.

## 1. Introduction

Removable and fixed dental prostheses are used for interim and long-term oral rehabilitation of completely and partially edentulous individuals [1,2]. Polymeric and metallic frameworks are used to construct prosthodontic appliances. Acrylic (polymethylmethacrylate; PMMA) remains the most popular, esthetic and least expensive option for the construction of complete dentures [3] and metallic frameworks offer improved strength and longevity in comparison [4]. Obturators, also mostly constructed of acrylics, are prosthodontic appliances constructed to occlude an oronasal fistula which may exist due to a cleft palate or surgery [5]. Outcomes of dental prosthetic treatment may be reported in the clinic (e.g., retention, occlusal stability, debonding of the base material from the framework, implantitis etc.) or by the patient (e.g., esthetics, masticatory function, fractures, etc.).

The materials used to produce these prosthodontic appliances have several disadvantages. Firstly, acrylics have limited strength and may undergo dimensional change during processing [3,6]. On the other hand, although metallic denture frameworks have improved mechanical properties, they are unesthetic and the metallic framework may debond from the overlying acrylic or porcelain [7,8]. Furthermore, acrylic and some metals may also cause allergic reactions in some individuals [9,10]. Moreover, long-span fixed porcelain-fused-to-metal (PFM) prostheses may fracture more easily and, therefore, are contraindicated in patients with inadequate or periodontally compromised abutment teeth [11]. Other major drawbacks of currently used materials are the long processing time and several appointments needed for the clinical procedures. Therefore, recent research was focused on finding a cost-effective alternative to these materials that is not only esthetic but offers more longevity.

The ideal prosthetic or implant material should be biocompatible, possess adequate mechanical properties to withstand occlusal forces, have favourable esthetic attributes and should not exert forces detrimental to the surrounding hard and soft tissues. Polyetheretherketone (PEEK) is a polymer produced by the step-growth dialkylation reaction of bis-phenolates [12]. PEEK was used for the construction of spinal fusion devices and other surgically placed implants [13]. In dentistry, the polymer was used in the construction of dental implants, orthodontic wires and dental prostheses [14]. Studies reporting the use of PEEK in spinal implants indicate that the material has excellent biocompatibility [13]. Because of their excellent mechanical strength, surface-modified PEEK implants were also studied for their potential to replace titanium as the material of choice [14]. Besides being esthetic, a major advantage of PEEK is that has physical properties comparable to that of human bone [15]. Therefore, it was suggested that PEEK appliances distribute forces more favourably than acrylics and metals [16]. More recently, modified forms of PEEK were produced that have antimicrobial and bioactive properties [17]. Given these favourable attributes, PEEK-based prosthodontic appliances [18] and obturators [19] were studied to overcome the drawbacks of conventional prosthodontic materials. There are several ways to process PEEK-based appliances. These include computer-aided design/computer-aided manufacture (CAD–CAM) workflows [20], conventional lost-wax casting [21] and injection moulding [22]. Of these processes, CAD–CAM PEEK prostheses have the unique advantage of being able to be constructed in a single appointment which is more convenient and time-saving for patients as well as the dental practitioner [23]. Studies also suggest that PEEK-based dental prostheses may have survival rates higher than 90%, which is comparable to that of other CAD–CAM materials such as titanium [24]. Moreover, in vitro laboratory studies have indicated that CAD–CAM dentures are more accurate and hence have a better fit when compared to conventionally processed prostheses [25]. However, to date, no systematic review has analysed the currently available evidence regarding the use of PEEK-based dental prostheses. Hence, the aim of this systematic review is to not only summarise the currently available evidence but also to critically analyse the literature that has focused on dental prostheses constructed with PEEK.

## 2. Materials and Methods

### 2.1. Focused Question and Protocol Registration

**Figure 1:**
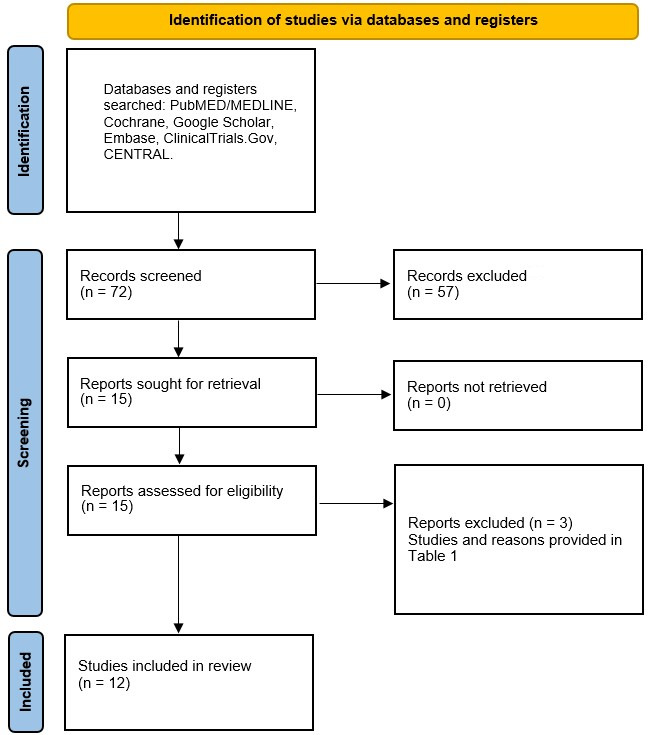
PRISMA flow diagram of the search methodology employed for this review.

Using the Participant, Intervention, Control and Outcomes (PICO) principle provided in the Preferred Reported Items for Systematic Reviews and Meta-analyses (PRISMA) statement in Figure 1 [26], the following focused question was constructed ‘Are dental prostheses made of PEEK inferior to those made of other materials in terms of clinical- and patient-reported outcomes?’. Outcomes such as implant-related complications, fractures, debonding of material stability were classified as clinical and those such as appearance and masticatory function were classified as patient-reported. The protocol for this review was registered on PROSPERO under the registration number CRD42021290311.

### 2.2. Eligibility Criteria

Prior to beginning the literature search, eligibility criteria for research pertinent to this review were established. Randomised controlled trials (RCT), cohort studies, case-control studies and case reports that focused on reporting clinical and patient-reported outcomes of dental prostheses constructed with PEEK frameworks or major connectors were included. Laboratory studies, animal studies, commentaries, reviews, letters to the editor and studies not in English were excluded. Excluded studies along with reasons for exclusion are listed in Table 1.

### 2.3. Literature Search

Three investigators (ZK, BMN and RM) conducted an electronic literature search via PubMED/MEDLINE, Google Scholar, EMBASE and ISI Web of Science using the following medical subject heading (MeSH) keywords: ((polyetheretherketone) OR (PEEK)) AND ((denture) OR (prosthodontic) OR (bridge) OR (denture framework) OR (dental prosthesis) OR (partial denture) OR (complete denture) OR (fixed denture) OR (removable dental prosthesis) OR (fixed dental prosthesis)) and the above-mentioned eligibility criteria for studies published between January 1990 and April 2022. Furthermore, with the assistance of the remaining two investigators (SB and SN), the reference lists in the complete texts of possibly eligible papers were examined to locate other studies that could fit the inclusion criteria.

### 2.4. Data Extraction

Using the PICO principle, the data from each study were independently extracted by the two investigators using a pre-decided data collection form. Any disagreements were solved by discussion. Briefly, data corresponding to the following categories was extracted: the type of study, number of patients, type or brand of PEEK, the mean age or range of the age of the patients, rehabilitation and study group details, the fabrication details, dental implant details (number and dimensions), duration of the studies (follow-up) and the outcomes. The data categories, along with extracted data, are listed in Table 2 and the outcomes are provided in Table 3.

### 2.5. Quality Assessment of Included Studies

For the quality assessment of the randomised clinical trials, the CONSORT tool [30] was used. STROBE quality assessment tool was used to assess the quality of observational studies [31] and the case reports were evaluated using the CARE guidelines [32]. Each study was given a relative grade of ‘low’, ‘medium’ and ‘high’ depending on the assessment criteria fulfilled by each study. The topics or sections evaluated in the included studies are presented in Table 4, Table 5 and Table 6.

## 3. Results

### 3.1. Results of the Literature Search

The initial search resulted in 72 items. In total, 57 irrelevant articles were excluded based on titles and abstracts and the full texts of 15 articles were downloaded to deem their eligibility for inclusion in this review. Of these 15 articles, three articles were excluded [27,28,29]. The reasons for their exclusion are provided in Table 1. Therefore, 12 studies were deemed suitable for inclusion in this review [20,24,33,34,35,36,37,38,39,40,41,42]. No additional studies were found upon hand searching, and none was found within the references of the included studies. The inter-examiner reliability (Cohen’s kappa) score was calculated as 0.83.

### 3.2. General Characteristics of Included Studies

Eight of the included studies were case reports [20,33,34,35,36,37,38,40], two studies were cohort studies [24,39], one study was a randomised controlled trial (RCT) [41] and another one was a case-control study [42]. Case reports documented six patients who received single PEEK prostheses [20,33,34,35,36,37,38,40]. The number of patients in the other studies ranged from 15 to 43 [24,39,41,42]. The age range of the patients ranged from 32 to 85 years [20,24,33,34,35,36,37,38,39,40,42]. The mean age was calculated as 59.96 years [20,24,33,34,35,36,37,38,39,40,42] while, in one study, the age of the patients was not reported [41]. BioHPP PEEK was used to construct prostheses in five studies [24,34,36,38,40]. PEEK Optima was used in the construction of prostheses in two studies [33,37] and Ceramill PEEK was also used in two studies [20,35]. While one study reported the use of the PEEK brand called Dental Direkt [41], two studies did not specify the type or brand of PEEK used [39,42]. In four studies, removable PEEK dentures were constructed [20,38,39,42] and fixed dental prostheses (FDP) were fabricated in three studies [24,36,37]. PEEK obturators were constructed in three studies [33,40,41] and PEEK was used to fabricate interim fixed in two studies [34,35]. In four studies, CAD–CAM was used for the construction of the prostheses [24,39,40,42] and in three studies, conventional impressions and lost-wax technique were used [36,37,38]. In one study, a PEEK obturator was constructed via the mechanical duplication of an older acrylic obturator [33]. In three studies, PEEK frameworks were digitally milled upon scanning of a wax pattern [20,34,35,41]. In two studies, conventional impressions were used in combination with CAM [20,41]. Implants were placed to support PEEK dentures in five studies [24,34,35,36,39] and the number of implants placed in each study ranged from 2 to 331 [24,34,35,36,39]. Among the five studies that had reported the use of implants, three studies reported the dimensions of the implants; the diameters ranged from 3.3 to 4.8 mm and the lengths ranged from 8 to 11 mm [34,36,39].

### 3.3. Outcomes of the Included Studies

In the case reports, PEEK dentures were successfully used for the oral rehabilitation of eight patients without any clinical or patient-reported complications [20,33,34,35,36,37,38,40]. In one cohort study, 20% of the implant-supported PEEK overdentures failed due to loss of passive fit, peri-implantitis developed in two patients and two dentures had to be repaired [39]. On the other hand, in a retrospective study, the 5-year survival rate of PEEK FDPs was reported to be 93.1%, which was statistically similar to the success rate of titanium FDPs which was reported to be 93.5% [24]. In the case-control study, in which the ridge changes of individuals who wore PEEK dentures were compared to those who did not wear any dentures, there was no difference observed between the outcomes of both the groups [42]. In the randomised controlled trial, attachment retained PEEK and metallic obturators exhibited similar bone loss and patient satisfaction but both the materials reported better outcomes when compared to conventional clasp-retained obturators [41]. In one study, 13.8% of the PEEK group and 16.1% of the titanium group exhibited bleeding on probing, soft tissue inflammation was observed in 3.4% of the PEEK group and 3.2% of the titanium prostheses, and temporomandibular disorders were observed in the 6.5% for the titanium group and none in the PEEK prostheses [24]. Furthermore, in the same study, PEEK prostheses resulted in significantly lesser (0.70 mm) vertical bone loss when compared to Ti, which resulted in 0.96 mm of vertical bone loss after 5 years [24].

### 3.4. Results of the Quality Assessment

Two case studies received an overall grade of medium [35,36] and the overall quality of six studies was graded as ‘low’ [20,33,34,37,38,40]. All three observational studies and the only RCT received scores of ‘medium’ [24,39,41,42]. The detailed results of the quality assessment are presented in Table 3, Table 4 and Table 5.

## 4. Discussion

Several materials are used in the construction of dental prostheses. Conventional materials include acrylics (with or without metallic frameworks), alloys and porcelain-fused-to-metal. However, studies suggested that these materials have a high percentage of failure or complications after five years of placement. Systematic review and meta-analysis of 32 studies have suggested that implant-supported fixed dental prostheses may have a failure rate of as high as 33.6% after 5 years [43]. The same study also concluded that the biggest cause of prostheses failure of implant-supported fixed dental prostheses is the fracture of the veneering material (13.5%) followed by peri-implantitis (8.5%) [43]. Conversely, conventional fixed bridges were reported to have a 15-year survival rate of 74% [44]. Acrylic partial dentures, primarily used as interim prostheses, were reported to last 6 to 12 months [45]. On the other hand, removable metal dentures were reported to have a 5-year survival rate of 75% [46]. Although alloy and PFM prostheses were constructed conventionally to overcome the limited strength and fracture resistance of acrylics, they are unable to prevent alveolar bone loss [47] most likely due to unfavourable stress distribution [48]. Although using dental implants to retain dental prostheses results in lesser bone resorption [49], it does not prevent it completely [50].

PEEK, with mechanical properties similar to that of cortical bone [15], is currently being studied as an alternative to conventional materials used in the fabrication of fixed and removable dental prostheses [20,24,33,34,35,36,37,38,39,40,41,42]. Indeed, PEEK’s flexural strength (183 MPa) was shown to be much higher than that of polymethylmethacrylate (PMMA; 84 MPa) in a recent in vitro investigation [51]. Similarly, in the case reports reviewed in this systematic review, favourable outcomes in the case reports reviewed in this systematic review suggest that PEEK is a promising material to replace PMMA as the material of choice to construct interim prostheses [34,35]. Nevertheless, a higher cost of PEEK may limit its clinical usage. Furthermore, a lack of randomised clinical trials focusing on PEEK dental prostheses indicates that there the evidence to use PEEK is inconclusive. Other materials such as base alloys and acrylics have been used for a longer time and, hence, are reported in the literature significantly more compared to PEEK.

Fractures at the acrylic–metal interface may account for up to 38% of denture fractures [52]. Perhaps the biggest advantage of PEEK frameworks that reinforce acrylic prostheses is that they may overcome the mismatch between the mechanical properties of metal frameworks and the acrylic components of conventional removable prostheses [20,38,39,42]. Moreover, recent research is being conducted to improve the bonding between PEEK and acrylic to further improve the durability of the PEEK–acrylic interface [53]. Therefore, PEEK–acrylic prostheses hold the potential for constructing durable and cost-effective dental prostheses. Due to PEEK’s tensile properties being similar to bone, mechanical properties superior to those of conventional acrylics and due to its non-brittle nature, PEEK-based prostheses supported by dental implants may potentially survive longer than conventional implant-supported dentures. Nevertheless, in the retrospective study by Wang et al., a 5-year survival rate of 93% for implant-supported was reported and none of the 331 implants placed failed [24]. Nevertheless, in the PEEK group, there was significantly lesser vertical peri-implant bone loss observed (0.70 mm) after 5 years compared to the same around the implants placed under titanium prostheses (096 mm) [24], which could be due to a reduced level of stress-shielding that was reported in prior finite element analysis (FEA) studies on PEEK prostheses [14]. In the same study, however, there was no significant difference observed between the survival of PEEK and Ti denture frameworks [24]. The results from this study suggest that for implant-supported prostheses, implant outcomes may determine the viability of both, PEEK and Ti denture frameworks [24]. In contrast, Mangano et al. reported a 20% prosthodontic failure rate in a prospective cohort trial and two of the 60 implants placed developed peri-implantitis [39]. Therefore, more long-term clinical trials are required to ascertain the survival of implant-supported PEEK dentures.

The studies included in this review had several limitations. A significant limitation is the lack of clinical trials comparing the survival of conventional prostheses with that of PEEK dental prostheses. Furthermore, most studies included in this review were case reports, and minimal sample sizes were included in those studies. Additionally, due to the nature of these studies, it was not possible to randomise the patients or blind the investigators. Therefore, the resulting bias may have influenced the outcomes of these studies. A major limitation of this systematic review was that it was not possible to conduct a meta-analysis due to the heterogeneous nature of the included studies and a lack of multiple clinical trials. Therefore, the currently available evidence is insufficient to gauge the durability and viability of PEEK-based dental prostheses. Moreover, none of the studies received a high score during the quality assessment, which further undermines the quality of the evidence presented in this systematic review.

## 5. Conclusions

Within the limitations of this study, the evidence to ascertain the long-term viability of PEEK-based dental prostheses is insufficient. The majority of the evidence regarding the outcomes of PEEK dental prostheses is obtained from case reports and non-randomised observational studies. Therefore, future studies should focus on conducting large-scale, multicenter trials to compare the survival rate of PEEK prostheses to that of conventionally available prosthodontic appliances. Additionally, implant-supported PEEK prostheses should be studied further for their potential to replace conventional materials and designs.

## Figures and Tables

**Table 1:** A list of the full texts excluded along with reasons for exclusion.

Study	Reason for Exclusion
**Ye et al. [27]**	Only workflow; PEEK denture not delivered to patient
**Yue et al. [28]**	PEEK framework not used
**Ichikawa [29]**	PEEK framework not used

**Table 2:** General characteristics of studies included in this review. PEEK: polyetheretherketone; N/A: not application; CAD: computer-aided design; CAM: computer-aided manufacture.

No.	Study—Author(s) and Year	Type of Study	Patient (s) (n)	Age (Mean/Range)	Type/Brand of PEEK	Rehabilitation Details and/or Study Groups	Fabrication	Implants Placed (n)	Implant Dimensions (mm)	Duration of Study
**1**	Costa-Palau et al., 2014	Case report	1	58 years	PEEK-Optima	Maxillary obturator.	Mechanical duplication of old obturator.	0	N/A	6 months
**2**	Zoidis and Papathanasiou, 2016	Case report	1	52 years	BioHPP PEEK	Interim fixed implant-supported 3-unit prosthesis.	Digital scanning of wax pattern and injection molding.	2	L = 11.5 D = 4	4 months
**3**	Hahnel et al., 2017	Case report	1	76 years	Ceramill PEEK	Interim maxillary all-on-four implant-supported PEEK fixed prosthesis.	Conventional wax pattern. CAM	4	NR	3 months
**4**	Zoidis 2017	Case report	1	65 years	BioHPP PEEK	Definitive maxillary fixed all-on-four implant-supported PEEK framework and PMMA base and veneers.	Conventional impression. Lost-wax and casting.	4	L = 11.5 D = 4	2 years
**5**	Sinha et al., 2017	Case report	1	32 years	PEEK-Optima	FPD. Upper and lower incisors replaced with canine–canine abutments. PEEK framework with resin composite veneers.	Conventional impression. Lost-wax and casting.	0	N/A	6 months
**6**	Zoidis 2018	Case report	1	85 years	BioHPP PEEK	Removable mandibular PEEK framework and PMMA base retained by high noble ball attachments on both canines.	Conventional impression. Lost-wax casting.	0	N/A	Not reported
**7**	Harb et al., 2018	Case report	1	56 years	CeraMill PEEK	Removable PEEK mandibular Kennedy class I framework and PMMA base to replace first and second molars.	Conventional impressions. Digital scanning of a wax pattern. CAM.	0	N/A	Not reported
**8**	Mangano et al., 2019	Prospective cohort	15	68.8 ± 4.7 years	Not recorded	Each patient received one removable maxillary overdenture supported by 4 implants and PEEK bar.	CAD–CAM replication of a relined denture. CAD: Meshmixer, Autodesk CAM: 3500 PD 3D printer, DWS	60	L = 8–14 D = 3.3–4.8	1 year
**9**	Tasopoulos et al., 2020	Case report	1	47 years	BioHPP	Two-piece PEEK maxillary obturator; Kennedy Class II (canine to second molar). Acrylic supported by PEEK framework.	Material: BioHPP Construction of 3D model using wax pattern. CAD: 3Shape Dental CAM: External laboratory	0	N/A	1 year
**10**	Wang et al., 2021	Retrospective cohort	43	59.8 years	BioHPP	Full-mouth FDP, 6 implants per arch (n = 60): Group I: PEEK framework and PMMA veneers (n = 29) Group II: Titanium framework and PMMA veneers (n = 31).	CAD: D2000 3D Scanner, 3Shape A/S CAM: 308 B, Willemin-Macodel (Ti), D3608, Sirona (PEEK) Dental Systems	331	N/A	5 years
**11**	Sharaf and Eskandar 2021	Randomised control trial	18	Not stated	Dental Direkt	Group I: Attachment-retained obturator with PEEK framework (n = 6) Group II: Attachment-retained obturator with metallic framework (n = 6) Group III: Conventional clasp-retained obturators with metallic framework (n = 6)	Conventional impressions. Digital scanning of wax pattern. CAM: Exocad GmbH	0	N/A	12 months
**12**	Russo et al., 2021	Case-control	16	46–72 years	Not specified	Group I: RPD (n = 10) Group II: Untreated partially edentulous (n = 6) Changes in residual ridge investigated for 1 year.	CAD: TRIOS 3, 3Shape A/S CAM: SmilesPeek	0	N/A	1 year

**Table 3:** Implant and prosthodontic outcomes of studies included in this review. PEEK: polyetheretherketone; BOP: bleeding on probing; TMJ: temporomandibular joint; VBL: vertical bone loss.

No.	Study—Author(s) and Year	Implant Outcomes	Prosthodontic Outcomes
**1**	Costa-Palau et al., 2014	No implants placed	No complications reported
**2**	Zoidis and Papathanasiou, 2016	No complications reported	No complications reported
**3**	Hahnel et al., 2017	No complications reported	No complications reported. OVD increased successfully on final follow-up
**4**	Zoidis 2017	No complications reported	No complications reported
**5**	Sinha et al., 2017	No implants placed	No complications reported
**6**	Zoidis 2018	No implants placed	No complications reported
**7**	Harb et al., 2018	No implants placed	No complications reported
**8**	Mangano et al., 2019	Peri-implantitis developed around 2 implants	20% of the dentures failed due to inadequate passive fit. 2 fractured dentures had to be repaired.
**9**	Tasopoulos et al., 2020	No implants placed	No complications reported
**10**	Wang et al., 2021	BOP: PEEK: 13.8%; Ti: 16.1% Soft tissue inflammationl: PEEK: 3.4%; Ti: 3.2% TMJ disorders: PEEK: None; Ti; 6.5% VBL: PEEK: 0.70 mm; Ti: 0.96 mm	5-year survival rate of PEEK and titanium overdentures comparable (93.1% and 93.5%, respectively).
**11**	Sharaf and Eskandar 2021	No implants placed	Group I and II exhibited lesser bone loss and greater patient satisfaction than Group III. No statistical difference between Groups I and II.
**12**	Russo et al., 2021	No implants placed	No significant differences between residual ridge changes in both groups

**Table 4:** Quality assessment results of the case reports included in this review.

**Study Characteristics**	**Study**							
**1.** **Title**	Costa-Palau et al., 2013	Zoidis and Papathanasiou, 2016	Hahnel et al., 2017	Zoidis 2017	Sinha et al., 2017	Zoidis 2017	Harb et al., 2018	Tasopoulos et al., 2020
**The diagnosis or intervention of primary focus followed by the words “case report”**	Yes	Yes	Yes	Yes	No	Yes	Yes	No
**2.** **Keywords**								
**2 to 5 keywords that identify diagnoses or interventions in this case report, including “case report”**	No	No	No	No	Yes	Yes	Yes	No
**3.** **Abstract**								
**Introduction**	No	No	Yes	Yes	Yes	Yes	No	No
**Symptoms/findings**	No	No	No	No	No	No	No	No
**Diagnoses and prosthodontic outcomes**	No	No	Yes	No	No	No	No	No
**Conclusions**	Yes	Yes	Yes	Yes	No	No	No	Yes
**4.** **Introduction**								
**One or two paragraphs summarising why this case is unique**	Yes	Yes	Yes	Yes	Yes	Yes	Yes	Yes
**5.** **Patient information**								
**De-identified patient information**	Yes	Yes	Yes	Yes	Yes	Yes	Yes	Yes
**Chief concerns and symptoms**	Yes	Yes	Yes	Yes	Yes	Yes	Yes	Yes
**Medical, family, psycho-social history, genetic information**	Yes	No	No	Yes	No	No	No	Yes
**Relevant past interventions with outcomes**	Yes	No	Yes	Yes	No	No	Yes	Yes
**6.** **Clinical findings**								
**Oral examination and important clinical findings**	Yes	Yes	Yes	Yes	Yes	Yes	Yes	Yes
**7.** **Timeline**								
**Historical and current information from this episode of care organised as a timeline**	No	No	No	No	No	No	No	No
**8.** **Diagnostic Assessment**								
**Oral examination**	Yes	Yes	Yes	Yes	Yes	Yes	Yes	Yes
**Previous denture history**	Yes	Yes	Yes	Yes	Yes	Yes	Yes	Yes
**Oral hygiene/periodontal status**	No	No	No	No	Yes	Yes	No	
**9.** **Prosthodontic rehabilitation**								
**Clinical procedures (impressions, intraoral scanning, surgery, etc.)**	Yes	Yes	Yes	Yes	Yes	Yes	Yes	Yes
**Laboratory procedures (wax-up, casting, fabrication, etc.)**	Yes	Yes	Yes	Yes	Yes	Yes	Yes	Yes
**Prosthesis design**	Yes	Yes	Yes	Yes	Yes	Yes	Yes	Yes
**10.** **Follow-up and outcomes**								
**Follow-up time period**	Yes	Yes	Yes	Yes	Yes	No	No	Yes
**Patient-reported outcomes**	Yes	Yes	Yes	Yes	No	No	Yes	No
**Prosthesis and/or implant outcomes**	Yes	Yes	Yes	Yes	Yes	No	Yes	Yes
**Complications/adverse effects**	No	Yes	Yes	Yes	Yes	No	No	No
**11.** **Discussion**								
**Strengths and limitations**	No	No	No	No	No	No	No	No
**Review of relevant literature**	No	Yes	Yes	No	No	Yes	Yes	Yes
**The scientific rationale for any conclusions**	Yes	Yes	Yes	Yes	No	Yes	Yes	Yes
**12.** **Conclusion**	Yes	Yes	Yes	Yes	Yes	Yes	Yes	Yes
**13.** **Informed consent**	No	No	No	No	No	No	No	No
**Overall quality**	Low	Low	Medium	Medium	Low	Low	Low	Low

**Table 5:** Quality assessment of the observational studies included in this review.

Section/Topic	Mangano et al., 2019	Wang et al., 2021	Russo et al., 2021
**1.** **Title and abstract**			
**Study design in title**	No	Yes	No
**Adequate abstract**	Yes	Yes	Yes
**2.** **Introduction**			
**Scientific background and rationale**	Yes	Yes	Yes
**State specific objectives and hypothesis**	Yes	Yes	Yes
**3.** **Methods**			
**Study design**	Yes	Yes	Yes
**Recruitment, exposure, follow-up, and data collection**	Yes	Yes	Yes
**Participants**			
**Eligibility criteria**	No	Yes	No
**Number of exposed and unexposed**	No	No	No
**Variables**			
**Adequate description of variables**	Yes	Yes	Yes
**Data measurement**			
**Sources and methods of measurement**	Yes	Yes	Yes
**Bias**			
**Methods to reduce bias (randomisation or blinding)**	No	No	No
**Study size**			
**Statistical calculation of sample size**	No	No	No
**Quantitative analysis**			
**Description of quantitative variables**	No	No	No
**Statistical methods**			
**Description of statistical methods**	Yes	Yes	Yes
**Subgroup analysis**	No	No	No
**Handling of missing data**	No	No	No
**Loss to follow-up**	No	No	No
**Sensitivity**	No	No	No
**4.** **Results**			
**Participants**			
**Number analysed**	Yes	Yes	Yes
**Reasons for drop-out**	No	No	No
**Flow-diagram for recruitment**	No	No	No
**Descriptive data**			
**Demographic, clinical and social data**	No	No	No
**Missing data**	No	No	No
**Follow-up time**	Yes	Yes	Yes
**Outcomes**			
**Outcome events or summary measures**	Yes	Yes	Yes
**Main results**			
**Confidence-interval**	Yes	Yes	No
**Category boundaries**	No	No	No
**Translation of relative to absolute risk**	Yes	No	No
**5.** **Discussion**			
**Key results**	Yes	Yes	Yes
**Limitations**	Yes	Yes	Yes
**Interpretation**	Yes	Yes	Yes
**Generalisability**	Yes	Yes	Yes
**6.** **Funding details**	Yes	Yes	Yes
**Overall quality**	Medium	Medium	Medium

**Table 6:** Quality assessment results of the quality assessment of the randomised controlled trial included in this review.

Section/Topic	Quality Assessment
**1.** **Title and abstract**	** Study: Sharaf and Eskandar 2021 **
**Identification as a randomised trial**	Yes
**Structured summary of the study**	Yes
**2.** **Introduction**	
**Scientific background and rationale**	Yes
**Specific objectives or hypotheses**	Yes
**3.** **Methods**	
**Description of trial design**	Yes
**Changes to methods**	No
**Eligibility criteria for participants**	Yes
**Settings and locations where the data were collected**	No
**The interventions for each group.**	Yes
**Primary and secondary outcome measures**	
**Any changes to trial outcomes**	No
**Sample size calculation**	Yes
**Method used to generate the random allocation sequence**	Yes
**Type of randomisation**	Yes
**Mechanism used to implement the random allocation sequence**	Yes
**Who generated the allocation sequence, who enrolled participants**	No
**Blinding of investigators**	Yes
**Description of the similarity of interventions**	No
**Appropriate statistics**	Yes
**4.** **Results**	
**Number analysed**	Yes
**Losses and exclusions**	Yes
**Dates of recruitment and follow up**	Yes
**Why the trial ended or was stopped**	No
**Demographic and clinical characteristics for each group**	No
**For each group, number of participants included in each analysis**	Yes
**Estimated effect size and its precision (such as 95% confidence interval)**	Yes
**Absolute and relative effect sizes**	No
**Subgroup analysis**	No
**Harms or unintended effects in each group**	No
**5.** **Discussion**	
**Trial limitations and addressing sources of potential bias**	No
**Generalisability of the trial findings**	Yes
**Interpretation consistent with results**	Yes
**6.** **Other information**	
**Registration number**	Yes
**Accessible protocol**	Yes
**Funding**	Yes
**Overall quality**	Medium

## Data Availability

Not applicable.

## References

[1] Chochlidakis K., Einarsdottir E., Tsigarida A., Papaspyridakos P., Romeo D., Barmak A.B., Ercoli C. (2020). Survival rates and prosthetic complications of implant fixed complete dental prostheses: An up to 5-year retrospective study. J. Prosthet. Dent..

[2] Cunningham J. (1993). Bond strength of denture teeth to acrylic bases. J. Dent..

[3] Meng T.R., Latta M.A. (2005). Physical properties of four acrylic denture base resins. J. Contemp. Dent. Pract..

[4] Shimizu H., Tsue F., Obukuro M., Kido H., Takahashi Y., Ohmura H. (2005). Fracture strength of metal-based complete maxillary dentures with a newly designed metal framework. Int. Chin. J. Dent..

[5] Kreeft A., Krap M., Wismeijer D., Speksnijder C., Smeele L., Bosch S., Muijen M., Balm A. (2012). Oral function after maxillectomy and reconstruction with an obturator. Int. J. Oral Maxillofac. Surg..

[6] Wong D.M., Cheng L.Y., Chow T., Clark R.K. (1999). Effect of processing method on the dimensional accuracy and water sorption of acrylic resin dentures. J. Prosthet. Dent..

[7] Al Jabbari Y.S., Zinelis S., Al Taweel S.M., Nagy W.W. (2016). The effect of artificial aging on the bond strength of heat-activated acrylic resin to surface-treated nickel-chromium-beryllium Alloy. Open Dent. J..

[8] Schweitzer D.M., Goldstein G.R., Ricci J.L., Silva N., Hittelman E.L. (2005). Comparison of bond strength of a pressed ceramic fused to metal versus feldspathic porcelain fused to metal. J. Prosthodont. Implant Esthet. Reconstr. Dent..

[9] Jorge J.H., Giampaolo E.T., Machado A.L., Vergani C.E. (2003). Cytotoxicity of denture base acrylic resins: A literature review. J. Prosthet. Dent..

[10] Spiechowicz E., Glantz P.O., Axell T., Grochowski P. (1999). A long-term follow-up of allergy to nickel among fixed prostheses wearers. Eur. J. Prosthodont. Restor. Dent..

[11] De Backer H., Van Maele G., De Moor N., Van den Berghe L. (2008). Long-term results of short-span versus long-span fixed dental prostheses: An up to 20-year retrospective study. Int. J. Prosthodont..

[12] Wang F., Roovers J. (1993). Functionalization of poly(aryl ether ether ketone) (PEEK): Synthesis and properties of aldehyde and carboxylic acid substituted PEEK. Macromolecules.

[13] Toth J.M., Wang M., Estes B., Scifert J.L., Seim H.B., Turner A.S. (2006). Polyetheretherketone as a biomaterial for spinal applications. Biomaterials.

[14] Najeeb S., Zafar M.S., Khurshid Z., Siddiqui F. (2016). Applications of polyetheretherketone (PEEK) in oral implantology and prosthodontics. J. Prosthodont. Res..

[15] SanSandler J., Werner P., Shaffer M.S., Demchuk V., Altstädt V., Windle A.H. (2002). Carbon-nanofibre-reinforced poly(ether ether ketone) composites. Compos. Part A Appl. Sci. Manuf..

[16] Sarot J.R., Contar C.M.M., de Cruz A.C.C., Magini R.d.S. (2010). Evaluation of the stress distribution in CFR-PEEK dental implants by the three-dimensional finite element method. J. Mater. Sci. Mater. Med..

[17] Najeeb S., Khurshid Z., Matinlinna J.P., Siddiqui F., Nassani M.Z., Baroudi K. (2015). Nanomodified Peek Dental Implants: Bioactive Composites and Surface Modification—A Review. Int. J. Dent..

[18] Muhsin S.A., Hatton P.V., Johnson A., Sereno N., Wood D.J. (2019). Determination of Polyetheretherketone (PEEK) mechanical properties as a denture material. Saudi Dent. J..

[19] Villefort R.F., Tribst J.P.M., Piva A.M.D.O.D., Borges A.L., Binda N.C., Ferreira C.E.D.A., Bottino M.A., Von Zeidler S.L.V. (2020). Stress distribution on different bar materials in implant-retained palatal obturator. PLoS ONE.

[20] Harb I.E., Abdel-Khalek E.A., Hegazy S.A. (2019). CAD/CAM Constructed Poly(etheretherketone) (PEEK) Framework of Kennedy Class I Removable Partial Denture: A Clinical Report. J. Prosthodont..

[21] Arnold C., Hey J., Schweyen R., Setz J.M. (2018). Accuracy of CAD-CAM-fabricated removable partial dentures. J. Prosthet. Dent..

[22] Golbang A., Mokhtari M., Harkin-Jones E., Archer E., McIlhagger A. (2021). Additive manufacturing and injection moulding of high-performance IF-WS 2/PEEK nanocomposites: A comparative study. Front. Mater..

[23] Papathanasiou I., Kamposiora P., Papavasiliou G., Ferrari M. (2020). The use of PEEK in digital prosthodontics: A narrative review. BMC Oral Health.

[24] Wang J., Wu P., Liu H.-L., Zhang L., Liu L.-P., Ma C.-F., Chen J.-H. (2021). Polyetheretherketone versus titanium CAD-CAM framework for implant-supported fixed complete dentures: A retrospective study with up to 5-year follow-up. J. Prosthodont. Res..

[25] Pereira A.L.C., de Medeiros A.K.B., de Sousa Santos K., de Almeida É.O., Barbosa G.A.S., Carreiro A.d.F.P. (2021). Accuracy of CAD-CAM systems for removable partial denture framework fabrication: A systematic review. J. Prosthet. Dent..

[26] Moher D., Altman D.G., Liberati A., Tetzlaff J. (2011). PRISMA statement. Epidemiology.

[27] Ye H., Wang Z., Sun Y., Zhou Y. (2021). Fully digital workflow for the design and manufacture of prostheses for maxillectomy defects. J. Prosthet. Dent..

[28] Yue Q., Yilmaz B., Abou-Ayash S., Zimmermann P., Brägger U., Schimmel M. (2020). Use of an attachment system with angulated abutments and polyetheretherketone inserts to retain a maxillary overdenture: A clinical report. J. Prosthet. Dent..

[29] Ichikawa T., Kurahashi K., Liu L., Matsuda T., Ishida Y. (2019). Use of a Polyetheretherketone Clasp Retainer for Removable Partial Denture: A Case Report. Dent. J..

[30] Moher D., Schulz K.F., Altman D.G., Consort G. (2001). The CONSORT Statement: Revised Recommendations for Improving the Quality of Reports of Parallel-Group Randomized Trials.

[31] Von Elm E., Altman D.G., Egger M., Pocock S.J., Gøtzsche P.C., Vandenbroucke J.P. (2007). The Strengthening the Reporting of Observational Studies in Epidemiology (STROBE) statement: Guidelines for reporting observational studies. Bull. World Health Organ..

[32] Riley D.S., Barber M.S., Kienle G.S., Aronson J.K., von Schoen-Angerer T., Tugwell P., Kiene H., Helfand M., Altman D.G., Sox H. (2017). CARE 2013 explanations and elaborations: Reporting guidelines for case reports. J. Clin. Epidemiol..

[33] Costa-Palau S., Torrents-Nicolas J., Barberà M.B.-D., Cabratosa-Termes J. (2014). Use of polyetheretherketone in the fabrication of a maxillary obturator prosthesis: A clinical report. J. Prosthet. Dent..

[34] Zoidis P., Papathanasiou I. (2016). Modified PEEK resin-bonded fixed dental prosthesis as an interim restoration after implant placement. J. Prosthet. Dent..

[35] Hahnel S., Scherl C., Rosentritt M. (2018). Interim rehabilitation of occlusal vertical dimension using a double-crown-retained removable dental prosthesis with polyetheretherketone framework. J. Prosthet. Dent..

[36] Zoidis P. (2018). The all-on-4 modified polyetheretherketone treatment approach: A clinical report. J. Prosthet. Dent..

[37] Sinha N., Gupta N., Reddy K.M., Shastry Y.M. (2017). Versatility of PEEK as a fixed partial denture framework. J. Indian Prosthodont. Soc..

[38] Zoidis P. (2018). Polyetheretherketone Overlay Prosthesis over High Noble Ball Attachments to Overcome Base Metal Sensitivity: A Clinical Report. J. Prosthodont..

[39] Mangano F., Mangano C., Margiani B., Admakin O. (2019). Combining Intraoral and Face Scans for the Design and Fabrication of Computer-Assisted Design/Computer-Assisted Manufacturing (CAD/CAM) Polyether-Ether-Ketone (PEEK) Implant-Supported Bars for Maxillary Overdentures. Scanning.

[40] Tasopoulos T., Chatziemmanouil D., Kouveliotis G., Karaiskou G., Wang J., Zoidis P. (2020). PEEK Maxillary Obturator Prosthesis Fabrication Using Intraoral Scanning, 3D Printing, and CAD/CAM. Int. J. Prosthodont..

[41] Sharaf M.Y., Eskander A.E. (2022). PEEK versus Metallic Attachment-Retained Obturators for Patient Satisfaction: A Randomized Controlled Trial. Eur. J. Dent..

[42] Lo Russo L., Chochlidakis K., Caradonna G., Molinelli F., Guida L., Ercoli C. (2021). Removable Partial Dentures with Polyetheretherketone Framework: The Influence on Residual Ridge Stability. J. Prosthodont. Implant Esthet. Reconstr. Dent..

[43] Pjetursson B.E., Thoma D., Jung R., Zwahlen M., Zembic A. (2012). A systematic review of the survival and complication rates of implant-supported fixed dental prostheses (FDPs) after a mean observation period of at least 5 years. Clin. Oral Implant. Res..

[44] Scurria M.S., Bader J.D., Shugars D.A. (1998). Meta-analysis of fixed partial denture survival: Prostheses and abutments. J. Prosthet. Dent..

[45] Walmsley A.D. (2003). Acrylic Partial Dentures. Dent. Update.

[46] Bergman B., Hugoson A., Olsson C.-O. (1995). A 25 year longitudinal study of patients treated with removable partial dentures. J. Oral Rehabil..

[47] Wyatt C.C. (1998). The effect of prosthodontic treatment on alveolar bone loss: A review of the literature. J. Prosthet. Dent..

[48] Jacobs R., van Steenberghe D., Nys M., Naert I. (1993). Maxillary bone resorption in patients with mandibular implant-supported overdentures or fixed prostheses. J. Prosthet. Dent..

[49] Lemos C.A.A., Nunes R.G., Santiago-Júnior J.F., Gomes J.M.D.L., Limirio J.P.J.O., Rosa C.D.D.R.D., Verri F.R., Pellizzer E.P. (2021). Are implant-supported removable partial dentures a suitable treatment for partially edentulous patients? A systematic review and meta-analysis. J. Prosthet. Dent..

[50] Alrajhi M., Askar O., Habib A., Elsyad M. (2020). Maxillary Bone Resorption with Conventional Dentures and Four-Implant–Supported Fixed Prosthesis Opposed by Distal-Extension Partial Dentures: A Preliminary 5-year Retrospective Study. Int. J. Oral Maxillofac. Implant..

[51] Mutneja P., Shrivastava S.P., Dable R., Raj A.N., Srivastava S.B., Haque M. (2021). Comparison of Mechanical Properties of PEEK and PMMA: An In Vitro Study. J. Contemp. Dent. Pract..

[52] Darbar U.R., Huggett R., Harrison A. (1994). Denture fracture—A survey. Br. Dent. J..

[53] Mayinger F., Fiebig M., Roos M., Eichberger M., Lümkemann N., Stawarczyk B. (2021). Bonding Behavior Between Polyetheretherketone and Polymethylmethacrylate Acrylic Denture Polymer. J. Adhes. Dent..

